# Betulin 3,28-di-*O*-tosyl­ate

**DOI:** 10.1107/S1600536814016602

**Published:** 2014-07-23

**Authors:** Uldis Peipiņš, Niks Freimanis, Dmitrijs Stepanovs, Anatoly Mishnev, Māris Turks

**Affiliations:** a Department of Material Science and Applied Chemistry, Riga Technical University, 3 P. Valdena Str., Riga LV-1007, Latvia; b Ogre State Gymnasium, 14 Meza prosp., LV-5001, Ogre, Latvia; c Latvian Institute of Organic Synthesis, 21 Aizkraukles Str., Riga LV-1006, Latvia

**Keywords:** crystal structure, hydrogen bonding, betulin 3,28-di-*O*-tosyl­ate, natural product

## Abstract

The title compound, C_44_H_62_O_6_S_2_ {systematic name: (1*R*,3a*S*,5a*R*,5b*R*,7a*R*,9*S*,11a*R*,11b*R*,13a*R*,13b*R*)-5a,5b,8,8,11a-penta­methyl-1-(prop-1-en-2-yl)-3a-[(tos­yloxy)meth­yl]icosa­hydro-1*H*-cyclo­penta­[*a*]chrysen-9-yl 4-methyl­benzene­sulfonate}, was obtained by tosyl­ation of naturally occurring betulin. All the cyclo­hexane rings adopt chair conformations and the cyclo­pentane ring adopts a twisted envelope conformation, with the C atom bearing the tosyl­methyl substituent forming the flap. In the crystal, mol­ecules form a three-dimensional network through multiple weak C—H⋯O hydrogen bonds.

## Related literature   

For the first synthesis of betulin 3,28-di-*O*-tosyl­ate, see: Anjaneyulu *et al.* (1980 ▶). For natural occurrence and isolation of betulin and related terpenoides, see: Krasutsky (2006 ▶). For the biological activity of natural and semisynthetic lupane terpenoides including betulin derivatives, see: Tolstikova *et al.* (2006*a* ▶,*b* ▶); Tundis *et al.* (2014 ▶). For some of the first crystal data for the betulin series, see: 3β-lup-20 (29)-ene-3,28-diol di­acetate (betulin 3,28-di-*O*-acetate; Abbot *et al.*, 1958 ▶). For other crystal structures of related betulin derivatives with substituents on the O atoms at C3 and C28, see: Kommera *et al.* (2010 ▶); Trishin *et al.* (2010 ▶); Boryczka *et al.* (2013 ▶). For recent crystal structures of betulin and its solvates, see: Drebushchak *et al.* (2013 ▶); Drebushchak *et al.* (2010 ▶); Boryczka *et al.* (2012 ▶). For standard bond lengths, see: Allen *et al.* (1987 ▶). The nature of hydrogen bonding is described by Gilli (2002 ▶).
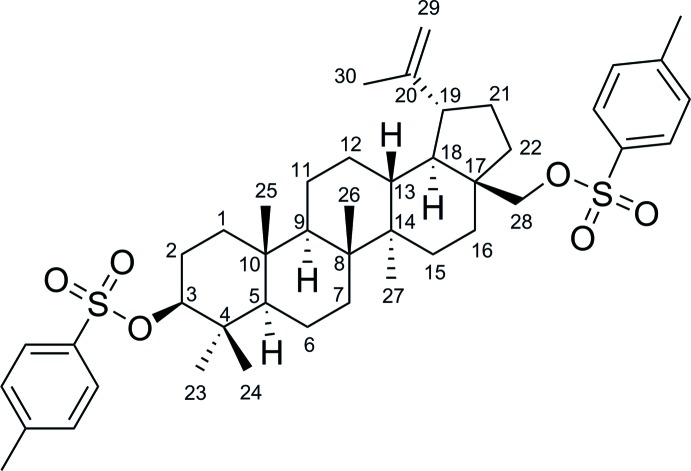


## Experimental   

### 

#### Crystal data  

C_44_H_62_O_6_S_2_
*M* *_r_* = 751.08
Orthorhombic, 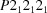

*a* = 6.9824 (1) Å
*b* = 18.2035 (4) Å
*c* = 31.4449 (9) Å
*V* = 3996.78 (16) Å^3^
*Z* = 4
Mo *K*α radiation
μ = 0.18 mm^−1^
*T* = 173 K
0.11 × 0.03 × 0.03 mm

#### Data collection  

Nonius KappaCCD diffractometer
9235 measured reflections
9235 independent reflections
4887 reflections with *I* > 2σ(*I*)

#### Refinement  

*R*[*F* ^2^ > 2σ(*F* ^2^)] = 0.077
*wR*(*F* ^2^) = 0.142
*S* = 1.01
9235 reflections
477 parameters
H-atom parameters constrained
Δρ_max_ = 0.26 e Å^−3^
Δρ_min_ = −0.33 e Å^−3^
Absolute structure: Flack (1983 ▶), 3968 Friedel pairs
Absolute structure parameter: 0.07 (9)

### 

Data collection: *KappaCCD Server Software* (Nonius, 1997 ▶); cell refinement: *SCALEPACK* (Otwinovski & Minor, 1997 ▶); data reduction: *DENZO* (Otwinovski & Minor, 1997 ▶) and *SCALEPACK*; program(s) used to solve structure: *SIR2011* (Burla *et al.*, 2012 ▶); program(s) used to refine structure: *SHELXL97* (Sheldrick, 2008 ▶); molecular graphics: *ORTEP-3 for Windows* (Farrugia, 2012 ▶); software used to prepare material for publication: *SHELXL97*, *PLATON* (Spek, 2009 ▶) and *publCIF* (Westrip, 2010 ▶).

## Supplementary Material

Crystal structure: contains datablock(s) I. DOI: 10.1107/S1600536814016602/fy2116sup1.cif

Structure factors: contains datablock(s) I. DOI: 10.1107/S1600536814016602/fy2116Isup2.hkl

Supporting information file. DOI: 10.1107/S1600536814016602/fy2116Isup3.cml

CCDC reference: 994542

Additional supporting information:  crystallographic information; 3D view; checkCIF report

## Figures and Tables

**Table 1:** Hydrogen-bond geometry (Å, °)

*D*—H⋯*A*	*D*—H	H⋯*A*	*D*⋯*A*	*D*—H⋯*A*
C21—H21*B*⋯O43^i^	0.97	2.60	3.428 (5)	143
C26—H26*A*⋯O32^ii^	0.96	2.56	3.473 (5)	159
C28—H28*A*⋯O43^i^	0.97	2.39	3.244 (5)	147
C48—H48⋯O44^iii^	0.93	2.49	3.142 (5)	128

Symmetry codes: (i) 
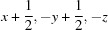
; (ii) 
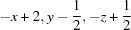
; (iii) 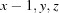.

**Fig. 1.**
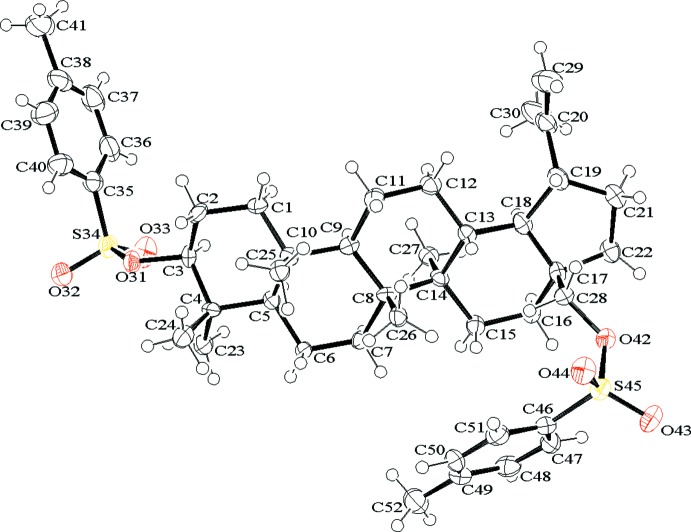
The asymmetric unit of the title compound showing 50% probability displacement ellipsoids and the atom-numbering. Hydrogen atoms are shown as small spheres of arbitrary radii.

## supplementary crystallographic information

### S1. Comment

The crystal and molecular structure of the betulin has not been previously reported, however, Drebushchak *et al.* (2013) have indexed X-ray powder diffraction pattern and calculated lattice parameters. Structures of the ethanol (Drebushchak *et al.*, 2010) and di­methyl sulfoxide (Boryczka *et al.*, 2012) solvates of betulin are known from single crystal X-ray diffraction data. Crystal structures of related betulin derivatives with substituents on oxygen atoms at C3 and C28 have been reported in literature, namely, 28-*O*-acetyl­betulin-3-yl-β-D-(2',3',4',6'-tetra-*O*-acetyl)­gluco­pyran­oside (Kommera *et al.*, 2010), betulin 3,28-di-*O*-tri­fluoro­acetate (Trishin *et al.*, 2010) and 28-*O*-propynoylbetulin di­methyl sulfoxide solvate (Boryczka *et al.*, 2013).

The bond lengths (Allen *et al.*, 1987) and angles in the molecule are close to standard values. All the cyclo­hexane rings adopt chair conformations and the cyclo­pentane ring adopts a twisted envelope conformation with the isopropenyl group equatorially attached to C19. The torsion angle C21—C19—C20—C29, which describs the conformation of the isopropenyl group, is equal to -102.1 (5)°. This conformation is variable among the structures disscused in this section. An *O*-tosyl group is attached to atom C3 in an equatorial orientation. The corresponding torsion angles C1—C2—C3—O31 and C1—C2—C3—O31 are -178.8 (3) and -179.2 (3)°, respectively. The *O*-tosyl­methyl group is is attached to the atom C17 in an axial orientation, with the corresponding torsion angles C13—C18—C17—C28 and C15—C16—C17—C28 being -57.9 (4) and 62.2 (4)°, respectively. All ring junctions in the structure are *trans*-fused. A similar conformation was observed in all structures mentioned in this section.

### S2. Experimental

Single crystals of betulin 3,28-di-*O*-tosyl­ate were grown from a hexanes/di­chloro­methane (15/1) solution by slow evaporation at ambient temperature. ^1^H-NMR and ^13^C-NMR spectra were recorded at 400 MHz and at 100.6 MHz, respectively. The proton signals for residual non-deuterated solvents (δ 7.26 for CDCl_3_) and carbon signals (δ 77.1 for CDCl_3_) were used as an inter­nal references for ^1^H-NMR and ^13^C-NMR spectra, respectively. Coupling constants are reported in Hz. Analytical thin layer chromatography (TLC) was performed on Kieselgel 60 F_254_ glass plates precoated with a 0.25 mm thickness of silica gel. Preparative flash chromatography was performed on silica gel (60 Å, 40-63 µm, ROCC). Melting points were recorded with a *Fisher Digital Melting Point Analyzer Model 355* apparatus. IR spectra were recorded in KBr with *FT—IR Perkin Elmer Spectrum BX*. Optical rotations were measured at 20 °C on a *Anton Paar MCP 500* polarimeter using a sodium lamp as the light source (589 nm). Dry pyridine was obtained by distillation over CaH_2_. Commercially available reagents were used as received.

Betulin 3,28-di-*O*-tosyl­ate. Tosyl chloride (1.08 g, 5.67 mmol, 2.50 equiv.) was added to a stirred solution of betulin (1.00 g, 2.26 mmol, 1.00 equiv.) and 4-di­methyl­amino­pyridine (DMAP; 25 mg, 0.2 mmol, 0.09 equiv.) in pyridine (10 mL) at ambient temperature. The resulting reaction mixture was stirred at ambient temperature for 72 h. Then pyridine was evaporated under reduced pressure keeping the water bath temperature below 35 °C. Toluene (10 mL) was added to the residue and the resulting mixture was evaporated under reduced pressure. Additional amount of toluene (2 × 10 mL) was added and the evaporation was repeated. This process removes the residual pyridine via azeotrope distillation. The resulting residue was directly transferred to silica gel column and chromatographed with EtOAc/hexanes (3/97). The fraction corresponding to R*_f_* =0.40 (EtOAc/hexanes 1:5) was collected and the obtained colorless powder (0.25 g, 15%) was crystallized from the hexanes/di­chloro­methane (15/1) solution by slow evaporation at ambient temperature to provide single crystals of betulin 3,28-di-*O*-tosyl­ate. Other fractions (1.10 g) contained the title product together with its mono-tosyl congeners.

Data for betulin 3,28-di-*O*-tosyl­ate: M.p. 130 °C (decomp.); [α]_D_^20^ = 24.2 (c = 0.40, CHCl_3_). IR (KBr), ν, cm^-1^: 2940, 2875, 1460, 1365, 1190, 1175, 1100, 960; ^1^H NMR (400 MHz, CDCl_3_), δ (ppm): 7.80 (d, 2H, ^3^*J*=7.8 Hz, H—C(Ar)), 7.78 (d, 2H, ^3^*J*=7.8 Hz, H—C(Ar)), 7.35 (d, 2H, ^3^*J*=7.8 Hz, H—C(Ar)), 7.31 (d, 2H, ^3^*J*=8.2 Hz, H—C(Ar)), 4.63 (bd, 1H, ^2^*J*=1.8 Hz, H_a_—C(29)), 4.57-4.56 (m, 1H, H_b_—C(29)), 4.19 (dd, 1H, ^3^*J*=11.7 Hz, ^3^*J*=5.1 Hz, H—C(3)), 4.05, 3.73 (2d, AB syst., 2H, ^2^*J*=9.4 Hz, H—C(28)), 2.45, 2.43 (2s, 6H, H_3_C-(Ts)), 2.27 (dt, 1H, ^3^*J*=11.0 Hz, ^3^*J*=5.6 Hz, H—C(19)), 1.85-0.64 (m, 42H; including: 1.63, 0.88 (2s, 6H); 0.781, 0.777, 0.773 (3s, 9H), 0.75 (s, 3H)); ^13^C NMR (100.6 MHz, CDCl_3_), δ (ppm): 149.6, 144.7, 144.2, 134.9, 132.8, 129.8 129.6, 128.0, 127.6, 110.1, 90.9, 69.2, 55.5, 50.0, 48.6, 47.6, 46.7, 42.5, 40.6, 38.6, 38.5, 37.6, 36.8, 34.1, 33.9, 29.2, 29.1, 27.8, 26.5, 24.9, 24.8, 21.6 (2C), 20.7, 19.0, 18.2, 16.2, 16.0, 15.7, 14.6.

### S3. Refinement

All non-hydrogen atoms were refined anisotropically. All hydrogen atoms were positioned geometrically with C—H distances ranging from 0.93 Å to 0.98 Å and refined as riding on their parent atoms with *U*_iso_ (H) = 1.5*U*_eq_ (C) for methyl groups and *U*_iso_ (H) = 1.2*U*_eq_ (C) for others.

There are 21 reflections with intensities affected by the beamstop; these were removed from the final refinement since they are in systematic error.

### Crystal data

**Table:**

C_44_H_62_O_6_S_2_	*D*_x_ = 1.248 Mg m^−^^3^
*M**_r_* = 751.08	Melting point: 403 K
Orthorhombic, *P*2_1_2_1_2_1_	Mo *K*α radiation, λ = 0.71073 Å
Hall symbol: P 2ac 2ab	Cell parameters from 35297 reflections
*a* = 6.9824 (1) Å	θ = 1.0–27.9°
*b* = 18.2035 (4) Å	µ = 0.18 mm^−^^1^
*c* = 31.4449 (9) Å	*T* = 173 K
*V* = 3996.78 (16) Å^3^	Needle, colorless
*Z* = 4	0.11 × 0.03 × 0.03 mm
*F*(000) = 1624	

### Data collection

**Table:**

Nonius KappaCCD diffractometer	4887 reflections with *I* > 2σ(*I*)
Radiation source: fine-focus sealed tube	θ_max_ = 27.8°, θ_min_ = 2.3°
Graphite monochromator	*h* = −9→9
CCD scans	*k* = −23→23
9235 measured reflections	*l* = −40→41
9235 independent reflections	

### Refinement

**Table:**

Refinement on *F*^2^	Secondary atom site location: difference Fourier map
Least-squares matrix: full	Hydrogen site location: inferred from neighbouring sites
*R*[*F*^2^ > 2σ(*F*^2^)] = 0.077	H-atom parameters constrained
*wR*(*F*^2^) = 0.142	*w* = 1/[σ^2^(*F*_o_^2^) + (0.0406*P*)^2^ + 1.1649*P*] where *P* = (*F*_o_^2^ + 2*F*_c_^2^)/3
*S* = 1.01	(Δ/σ)_max_ < 0.001
9235 reflections	Δρ_max_ = 0.26 e Å^−^^3^
477 parameters	Δρ_min_ = −0.33 e Å^−^^3^
0 restraints	Absolute structure: Flack (1983), 3968 Friedel pairs
0 constraints	Absolute structure parameter: 0.07 (9)
Primary atom site location: structure-invariant direct methods	

### Special details

**Table:**

Geometry. All e.s.d.'s (except the e.s.d. in the dihedral angle between two l.s. planes) are estimated using the full covariance matrix. The cell e.s.d.'s are taken into account individually in the estimation of e.s.d.'s in distances, angles and torsion angles; correlations between e.s.d.'s in cell parameters are only used when they are defined by crystal symmetry. An approximate (isotropic) treatment of cell e.s.d.'s is used for estimating e.s.d.'s involving l.s. planes.
Refinement. Refinement of *F*^2^ against ALL reflections. The weighted *R*-factor *wR* and goodness of fit *S* are based on *F*^2^, conventional *R*-factors *R* are based on *F*, with *F* set to zero for negative *F*^2^. The threshold expression of *F*^2^ > σ(*F*^2^) is used only for calculating *R*-factors(gt) *etc*. and is not relevant to the choice of reflections for refinement. *R*-factors based on *F*^2^ are statistically about twice as large as those based on *F*, and *R*- factors based on ALL data will be even larger.

### Fractional atomic coordinates and isotropic or equivalent isotropic displacement parameters (Å^2^)

**Table:**

	*x*	*y*	*z*	*U*_iso_*/*U*_eq_	
C1	1.2440 (5)	0.7646 (2)	0.13908 (14)	0.0270 (10)	
H1A	1.3758	0.7596	0.1297	0.032*	
H1B	1.1724	0.7868	0.1160	0.032*	
C2	1.2389 (5)	0.8165 (2)	0.17714 (14)	0.0299 (11)	
H2B	1.2816	0.8650	0.1686	0.036*	
H2A	1.3246	0.7986	0.1991	0.036*	
C3	1.0351 (5)	0.8208 (2)	0.19443 (13)	0.0262 (10)	
H3	0.9515	0.8404	0.1721	0.031*	
C4	0.9545 (5)	0.7472 (2)	0.20942 (12)	0.0211 (9)	
C5	0.9623 (5)	0.6960 (2)	0.16969 (13)	0.0203 (9)	
H5	0.8818	0.7204	0.1484	0.024*	
C6	0.8693 (5)	0.6208 (2)	0.17625 (13)	0.0204 (10)	
H6A	0.9549	0.5896	0.1925	0.024*	
H6B	0.7516	0.6265	0.1923	0.024*	
C7	0.8261 (5)	0.5848 (2)	0.13390 (13)	0.0248 (11)	
H7B	0.7291	0.6135	0.1194	0.030*	
H7A	0.7730	0.5363	0.1391	0.030*	
C8	1.0016 (5)	0.5769 (2)	0.10437 (13)	0.0206 (9)	
C9	1.1168 (5)	0.6507 (2)	0.10316 (13)	0.0213 (10)	
H9	1.0315	0.6857	0.0889	0.026*	
C10	1.1617 (5)	0.6869 (2)	0.14760 (13)	0.0214 (10)	
C11	1.2908 (5)	0.6442 (2)	0.07366 (14)	0.0286 (11)	
H11A	1.3505	0.6921	0.0711	0.034*	
H11B	1.3833	0.6113	0.0866	0.034*	
C12	1.2429 (5)	0.6157 (2)	0.02906 (14)	0.0277 (11)	
H12A	1.3611	0.6054	0.0140	0.033*	
H12B	1.1753	0.6537	0.0135	0.033*	
C13	1.1188 (5)	0.5455 (2)	0.02990 (13)	0.0222 (10)	
H13	1.1939	0.5078	0.0446	0.027*	
C14	0.9334 (5)	0.5586 (2)	0.05728 (13)	0.0227 (10)	
C15	0.8006 (5)	0.4902 (2)	0.05702 (14)	0.0262 (11)	
H15A	0.8543	0.4537	0.0760	0.031*	
H15B	0.6769	0.5044	0.0684	0.031*	
C16	0.7695 (5)	0.4547 (2)	0.01360 (13)	0.0259 (10)	
H16A	0.6900	0.4866	−0.0037	0.031*	
H16B	0.7018	0.4086	0.0173	0.031*	
C17	0.9584 (5)	0.4402 (2)	−0.00960 (13)	0.0249 (10)	
C18	1.0655 (5)	0.5138 (2)	−0.01344 (13)	0.0259 (10)	
H18	0.9758	0.5486	−0.0264	0.031*	
C19	1.2249 (6)	0.4991 (2)	−0.04710 (14)	0.0289 (11)	
H19	1.3344	0.4768	−0.0325	0.035*	
C20	1.2967 (6)	0.5640 (2)	−0.07193 (14)	0.0341 (12)	
C21	1.1336 (5)	0.4397 (3)	−0.07636 (14)	0.0327 (11)	
H21A	1.1122	0.4596	−0.1046	0.039*	
H21B	1.2183	0.3976	−0.0788	0.039*	
C22	0.9419 (5)	0.4165 (2)	−0.05622 (13)	0.0296 (11)	
H22A	0.8354	0.4410	−0.0700	0.036*	
H22B	0.9238	0.3638	−0.0584	0.036*	
C23	0.7438 (5)	0.7597 (2)	0.22178 (14)	0.0341 (11)	
H23A	0.7357	0.7993	0.2419	0.051*	
H23B	0.6932	0.7157	0.2343	0.051*	
H23C	0.6711	0.7719	0.1969	0.051*	
C24	1.0565 (6)	0.7175 (2)	0.24903 (15)	0.0355 (12)	
H24A	1.1923	0.7170	0.2442	0.053*	
H24B	1.0131	0.6684	0.2547	0.053*	
H24C	1.0281	0.7483	0.2730	0.053*	
C25	1.3102 (5)	0.6440 (2)	0.17375 (14)	0.0272 (11)	
H25A	1.4078	0.6253	0.1552	0.041*	
H25B	1.2483	0.6038	0.1880	0.041*	
H25C	1.3671	0.6760	0.1944	0.041*	
C26	1.1239 (5)	0.5136 (2)	0.12257 (13)	0.0259 (10)	
H26A	1.1191	0.5149	0.1531	0.039*	
H26B	1.2541	0.5192	0.1133	0.039*	
H26C	1.0744	0.4675	0.1127	0.039*	
C27	0.8145 (5)	0.6219 (2)	0.03740 (14)	0.0260 (11)	
H27A	0.7612	0.6059	0.0108	0.039*	
H27B	0.8959	0.6636	0.0326	0.039*	
H27C	0.7129	0.6353	0.0564	0.039*	
C28	1.0801 (5)	0.3841 (2)	0.01470 (14)	0.0288 (11)	
H28A	1.2077	0.3819	0.0026	0.035*	
H28B	1.0912	0.3986	0.0443	0.035*	
C29	1.4816 (7)	0.5723 (3)	−0.08026 (15)	0.0550 (15)	
H29A	1.5694	0.5376	−0.0707	0.066*	
H19B	1.5235	0.6128	−0.0957	0.066*	
C30	1.1497 (7)	0.6159 (3)	−0.09078 (17)	0.0626 (17)	
H30A	1.2127	0.6505	−0.1091	0.094*	
H30B	1.0858	0.6419	−0.0683	0.094*	
H30C	1.0576	0.5884	−0.1069	0.094*	
O31	1.0355 (4)	0.87278 (14)	0.23064 (9)	0.0304 (7)	
O32	0.8911 (4)	0.96763 (17)	0.27028 (11)	0.0481 (9)	
O33	0.7673 (4)	0.94160 (18)	0.19878 (11)	0.0491 (9)	
S34	0.92414 (15)	0.94805 (6)	0.22731 (4)	0.0342 (3)	
C35	1.0920 (6)	1.0093 (2)	0.20621 (15)	0.0318 (11)	
C36	1.0496 (7)	1.0516 (3)	0.17126 (15)	0.0446 (13)	
H36	0.9353	1.0441	0.1567	0.054*	
C37	1.1759 (9)	1.1051 (3)	0.15772 (18)	0.0576 (16)	
H37	1.1445	1.1343	0.1345	0.069*	
C38	1.3481 (9)	1.1156 (3)	0.17832 (19)	0.0520 (15)	
C39	1.3917 (7)	1.0712 (3)	0.21182 (18)	0.0509 (15)	
H39	1.5096	1.0767	0.2252	0.061*	
C40	1.2667 (6)	1.0181 (3)	0.22667 (16)	0.0406 (12)	
H40	1.2990	0.9889	0.2499	0.049*	
C41	1.4852 (10)	1.1756 (3)	0.16420 (19)	0.087 (2)	
H41A	1.4738	1.2170	0.1829	0.131*	
H41B	1.4543	1.1904	0.1357	0.131*	
H41C	1.6141	1.1573	0.1650	0.131*	
O42	0.9875 (4)	0.31153 (15)	0.01169 (9)	0.0296 (7)	
O43	0.9399 (4)	0.18894 (16)	0.03501 (10)	0.0404 (8)	
O44	1.2059 (3)	0.26027 (16)	0.06542 (9)	0.0358 (8)	
S45	1.01383 (14)	0.25713 (6)	0.04993 (4)	0.0301 (3)	
C46	0.8623 (6)	0.2946 (2)	0.08920 (14)	0.0289 (11)	
C47	0.6685 (6)	0.3034 (2)	0.08074 (15)	0.0317 (12)	
H47	0.6167	0.2866	0.0553	0.038*	
C48	0.5538 (6)	0.3373 (2)	0.11046 (15)	0.0355 (12)	
H48	0.4243	0.3441	0.1047	0.043*	
C49	0.6285 (6)	0.3617 (2)	0.14898 (15)	0.0324 (12)	
C50	0.8201 (6)	0.3507 (3)	0.15726 (14)	0.0326 (11)	
H50	0.8705	0.3656	0.1832	0.039*	
C51	0.9387 (6)	0.3177 (2)	0.12759 (14)	0.0323 (11)	
H51	1.0682	0.3112	0.1334	0.039*	
C52	0.5011 (7)	0.3981 (3)	0.18128 (15)	0.0469 (13)	
H52A	0.3703	0.3847	0.1760	0.070*	
H52B	0.5146	0.4504	0.1792	0.070*	
H52C	0.5372	0.3824	0.2093	0.070*	

### Atomic displacement parameters (Å^2^)

**Table:**

	*U*^11^	*U*^22^	*U*^33^	*U*^12^	*U*^13^	*U*^23^
C1	0.023 (2)	0.028 (3)	0.030 (3)	−0.0074 (19)	0.0076 (19)	−0.001 (2)
C2	0.025 (2)	0.026 (3)	0.039 (3)	−0.0111 (19)	0.002 (2)	−0.007 (2)
C3	0.032 (2)	0.024 (2)	0.023 (3)	0.0012 (19)	−0.003 (2)	−0.009 (2)
C4	0.020 (2)	0.024 (2)	0.019 (2)	−0.0028 (17)	0.0033 (16)	0.004 (2)
C5	0.017 (2)	0.025 (2)	0.019 (2)	0.0004 (17)	−0.0033 (17)	−0.001 (2)
C6	0.021 (2)	0.019 (2)	0.021 (3)	−0.0012 (17)	0.0040 (18)	−0.002 (2)
C7	0.022 (2)	0.023 (2)	0.030 (3)	−0.0030 (17)	0.001 (2)	−0.001 (2)
C8	0.018 (2)	0.020 (2)	0.023 (2)	0.0004 (17)	0.0017 (19)	0.0057 (19)
C9	0.017 (2)	0.019 (2)	0.028 (3)	0.0028 (17)	0.0007 (18)	0.002 (2)
C10	0.017 (2)	0.022 (2)	0.025 (3)	0.0034 (17)	0.0021 (18)	−0.002 (2)
C11	0.021 (2)	0.031 (3)	0.034 (3)	−0.0059 (18)	0.004 (2)	−0.006 (2)
C12	0.022 (2)	0.032 (3)	0.029 (3)	−0.001 (2)	0.002 (2)	0.000 (2)
C13	0.023 (2)	0.024 (2)	0.020 (2)	0.0036 (18)	0.0018 (18)	0.000 (2)
C14	0.018 (2)	0.028 (2)	0.022 (2)	−0.0002 (17)	0.0049 (18)	−0.001 (2)
C15	0.018 (2)	0.031 (3)	0.030 (3)	−0.0002 (17)	0.0038 (19)	−0.008 (2)
C16	0.028 (2)	0.025 (2)	0.025 (3)	−0.0002 (19)	−0.0016 (19)	−0.003 (2)
C17	0.021 (2)	0.032 (3)	0.022 (3)	0.0029 (18)	−0.0023 (18)	−0.008 (2)
C18	0.028 (2)	0.027 (3)	0.023 (3)	0.0101 (18)	0.000 (2)	0.000 (2)
C19	0.029 (2)	0.035 (3)	0.023 (3)	0.0067 (19)	−0.002 (2)	0.001 (2)
C20	0.046 (3)	0.034 (3)	0.022 (3)	0.004 (2)	0.007 (2)	0.002 (2)
C21	0.034 (2)	0.041 (3)	0.023 (3)	0.006 (2)	0.001 (2)	−0.003 (2)
C22	0.026 (2)	0.037 (3)	0.026 (3)	0.0029 (18)	−0.004 (2)	−0.004 (2)
C23	0.034 (2)	0.032 (3)	0.036 (3)	−0.004 (2)	0.013 (2)	−0.011 (2)
C24	0.043 (3)	0.028 (3)	0.035 (3)	−0.006 (2)	−0.002 (2)	−0.001 (2)
C25	0.023 (2)	0.030 (3)	0.028 (3)	−0.0003 (18)	−0.002 (2)	−0.002 (2)
C26	0.034 (2)	0.021 (2)	0.023 (3)	0.0017 (19)	0.002 (2)	−0.002 (2)
C27	0.020 (2)	0.031 (3)	0.027 (3)	0.0007 (18)	0.0012 (19)	0.000 (2)
C28	0.030 (2)	0.029 (3)	0.027 (3)	−0.010 (2)	0.004 (2)	−0.003 (2)
C29	0.058 (3)	0.069 (4)	0.038 (3)	−0.014 (3)	0.005 (3)	0.014 (3)
C30	0.087 (4)	0.057 (4)	0.044 (4)	0.026 (3)	0.024 (3)	0.025 (3)
O31	0.0409 (17)	0.0240 (16)	0.0263 (18)	−0.0003 (13)	−0.0001 (14)	−0.0059 (15)
O32	0.060 (2)	0.043 (2)	0.041 (2)	−0.0113 (16)	0.0168 (18)	−0.0135 (19)
O33	0.0344 (17)	0.048 (2)	0.065 (3)	0.0101 (16)	−0.0103 (17)	−0.015 (2)
S34	0.0377 (7)	0.0292 (7)	0.0358 (8)	0.0009 (5)	0.0062 (6)	−0.0095 (6)
C35	0.038 (3)	0.026 (3)	0.032 (3)	0.002 (2)	0.007 (2)	−0.004 (2)
C36	0.068 (3)	0.038 (3)	0.028 (3)	0.003 (3)	−0.001 (3)	−0.003 (3)
C37	0.107 (5)	0.036 (4)	0.029 (3)	0.003 (3)	0.003 (3)	0.005 (3)
C38	0.086 (4)	0.027 (3)	0.043 (4)	−0.003 (3)	0.031 (3)	−0.004 (3)
C39	0.047 (3)	0.044 (3)	0.062 (4)	−0.009 (3)	0.016 (3)	−0.008 (3)
C40	0.044 (3)	0.034 (3)	0.043 (3)	−0.001 (2)	0.004 (3)	0.006 (3)
C41	0.146 (6)	0.043 (3)	0.072 (5)	−0.037 (4)	0.054 (5)	−0.014 (3)
O42	0.0373 (15)	0.0265 (16)	0.0249 (17)	−0.0048 (13)	−0.0001 (14)	−0.0024 (15)
O43	0.0462 (18)	0.0261 (18)	0.049 (2)	−0.0067 (14)	−0.0029 (16)	−0.0075 (17)
O44	0.0289 (16)	0.0369 (19)	0.042 (2)	0.0036 (13)	−0.0039 (14)	−0.0044 (18)
S45	0.0314 (6)	0.0268 (6)	0.0321 (7)	−0.0035 (5)	−0.0012 (5)	−0.0027 (6)
C46	0.033 (3)	0.027 (3)	0.027 (3)	−0.0076 (19)	0.004 (2)	0.002 (2)
C47	0.029 (2)	0.038 (3)	0.029 (3)	−0.009 (2)	0.000 (2)	−0.006 (2)
C48	0.024 (2)	0.042 (3)	0.041 (3)	−0.007 (2)	0.000 (2)	0.005 (3)
C49	0.035 (3)	0.033 (3)	0.030 (3)	−0.006 (2)	0.007 (2)	0.003 (2)
C50	0.043 (3)	0.037 (3)	0.018 (3)	−0.006 (2)	−0.003 (2)	−0.001 (2)
C51	0.037 (3)	0.032 (3)	0.028 (3)	−0.006 (2)	−0.006 (2)	0.006 (2)
C52	0.048 (3)	0.056 (3)	0.037 (3)	−0.007 (3)	0.005 (3)	−0.004 (3)

### Geometric parameters (Å, º)

**Table:**

C1—C2	1.525 (6)	C22—H22B	0.9700
C1—C10	1.550 (5)	C23—H23A	0.9600
C1—H1A	0.9700	C23—H23B	0.9600
C1—H1B	0.9700	C23—H23C	0.9600
C2—C3	1.526 (5)	C24—H24A	0.9600
C2—H2B	0.9700	C24—H24B	0.9600
C2—H2A	0.9700	C24—H24C	0.9600
C3—O31	1.481 (4)	C25—H25A	0.9600
C3—C4	1.527 (5)	C25—H25B	0.9600
C3—H3	0.9800	C25—H25C	0.9600
C4—C24	1.534 (5)	C26—H26A	0.9600
C4—C23	1.538 (5)	C26—H26B	0.9600
C4—C5	1.560 (5)	C26—H26C	0.9600
C5—C6	1.530 (5)	C27—H27A	0.9600
C5—C10	1.565 (5)	C27—H27B	0.9600
C5—H5	0.9800	C27—H27C	0.9600
C6—C7	1.515 (5)	C28—O42	1.474 (5)
C6—H6A	0.9700	C28—H28A	0.9700
C6—H6B	0.9700	C28—H28B	0.9700
C7—C8	1.544 (5)	C29—H29A	0.9300
C7—H7B	0.9700	C29—H19B	0.9300
C7—H7A	0.9700	C30—H30A	0.9600
C8—C26	1.543 (5)	C30—H30B	0.9600
C8—C9	1.567 (5)	C30—H30C	0.9600
C8—C14	1.591 (5)	O31—S34	1.579 (3)
C9—C11	1.533 (5)	O32—S34	1.416 (3)
C9—C10	1.577 (6)	O33—S34	1.421 (3)
C9—H9	0.9800	S34—C35	1.748 (4)
C10—C25	1.537 (5)	C35—C36	1.374 (6)
C11—C12	1.532 (6)	C35—C40	1.388 (6)
C11—H11A	0.9700	C36—C37	1.381 (7)
C11—H11B	0.9700	C36—H36	0.9300
C12—C13	1.544 (5)	C37—C38	1.379 (7)
C12—H12A	0.9700	C37—H37	0.9300
C12—H12B	0.9700	C38—C39	1.363 (7)
C13—C18	1.526 (5)	C38—C41	1.519 (7)
C13—C14	1.573 (5)	C39—C40	1.383 (6)
C13—H13	0.9800	C39—H39	0.9300
C14—C27	1.551 (5)	C40—H40	0.9300
C14—C15	1.552 (5)	C41—H41A	0.9600
C15—C16	1.527 (5)	C41—H41B	0.9600
C15—H15A	0.9700	C41—H41C	0.9600
C15—H15B	0.9700	O42—S45	1.569 (3)
C16—C17	1.530 (5)	O43—S45	1.424 (3)
C16—H16A	0.9700	O44—S45	1.428 (3)
C16—H16B	0.9700	S45—C46	1.763 (4)
C17—C22	1.533 (5)	C46—C51	1.386 (6)
C17—C28	1.533 (6)	C46—C47	1.388 (6)
C17—C18	1.539 (5)	C47—C48	1.377 (6)
C18—C19	1.559 (5)	C47—H47	0.9300
C18—H18	0.9800	C48—C49	1.391 (6)
C19—C20	1.502 (6)	C48—H48	0.9300
C19—C21	1.557 (6)	C49—C50	1.377 (5)
C19—H19	0.9800	C49—C52	1.504 (6)
C20—C29	1.326 (6)	C50—C51	1.384 (6)
C20—C30	1.516 (6)	C50—H50	0.9300
C21—C22	1.540 (5)	C51—H51	0.9300
C21—H21A	0.9700	C52—H52A	0.9600
C21—H21B	0.9700	C52—H52B	0.9600
C22—H22A	0.9700	C52—H52C	0.9600
			
C2—C1—C10	114.9 (3)	C22—C21—H21A	110.2
C2—C1—H1A	108.6	C19—C21—H21A	110.2
C10—C1—H1A	108.6	C22—C21—H21B	110.2
C2—C1—H1B	108.6	C19—C21—H21B	110.2
C10—C1—H1B	108.6	H21A—C21—H21B	108.5
H1A—C1—H1B	107.5	C17—C22—C21	104.5 (3)
C1—C2—C3	109.5 (3)	C17—C22—H22A	110.8
C1—C2—H2B	109.8	C21—C22—H22A	110.8
C3—C2—H2B	109.8	C17—C22—H22B	110.8
C1—C2—H2A	109.8	C21—C22—H22B	110.8
C3—C2—H2A	109.8	H22A—C22—H22B	108.9
H2B—C2—H2A	108.2	C4—C23—H23A	109.5
O31—C3—C2	107.8 (3)	C4—C23—H23B	109.5
O31—C3—C4	108.9 (3)	H23A—C23—H23B	109.5
C2—C3—C4	114.1 (3)	C4—C23—H23C	109.5
O31—C3—H3	108.6	H23A—C23—H23C	109.5
C2—C3—H3	108.6	H23B—C23—H23C	109.5
C4—C3—H3	108.6	C4—C24—H24A	109.5
C3—C4—C24	112.9 (3)	C4—C24—H24B	109.5
C3—C4—C23	107.5 (3)	H24A—C24—H24B	109.5
C24—C4—C23	106.9 (3)	C4—C24—H24C	109.5
C3—C4—C5	105.3 (3)	H24A—C24—H24C	109.5
C24—C4—C5	115.0 (3)	H24B—C24—H24C	109.5
C23—C4—C5	108.9 (3)	C10—C25—H25A	109.5
C6—C5—C4	114.4 (3)	C10—C25—H25B	109.5
C6—C5—C10	110.0 (3)	H25A—C25—H25B	109.5
C4—C5—C10	116.7 (3)	C10—C25—H25C	109.5
C6—C5—H5	104.8	H25A—C25—H25C	109.5
C4—C5—H5	104.8	H25B—C25—H25C	109.5
C10—C5—H5	104.8	C8—C26—H26A	109.5
C7—C6—C5	110.7 (3)	C8—C26—H26B	109.5
C7—C6—H6A	109.5	H26A—C26—H26B	109.5
C5—C6—H6A	109.5	C8—C26—H26C	109.5
C7—C6—H6B	109.5	H26A—C26—H26C	109.5
C5—C6—H6B	109.5	H26B—C26—H26C	109.5
H6A—C6—H6B	108.1	C14—C27—H27A	109.5
C6—C7—C8	114.2 (3)	C14—C27—H27B	109.5
C6—C7—H7B	108.7	H27A—C27—H27B	109.5
C8—C7—H7B	108.7	C14—C27—H27C	109.5
C6—C7—H7A	108.7	H27A—C27—H27C	109.5
C8—C7—H7A	108.7	H27B—C27—H27C	109.5
H7B—C7—H7A	107.6	O42—C28—C17	108.8 (3)
C26—C8—C7	106.6 (3)	O42—C28—H28A	109.9
C26—C8—C9	111.4 (3)	C17—C28—H28A	109.9
C7—C8—C9	110.0 (3)	O42—C28—H28B	109.9
C26—C8—C14	110.8 (3)	C17—C28—H28B	109.9
C7—C8—C14	110.0 (3)	H28A—C28—H28B	108.3
C9—C8—C14	108.1 (3)	C20—C29—H29A	120.0
C11—C9—C8	110.8 (3)	C20—C29—H19B	120.0
C11—C9—C10	114.3 (3)	H29A—C29—H19B	120.0
C8—C9—C10	116.0 (3)	C20—C30—H30A	109.5
C11—C9—H9	104.8	C20—C30—H30B	109.5
C8—C9—H9	104.8	H30A—C30—H30B	109.5
C10—C9—H9	104.8	C20—C30—H30C	109.5
C25—C10—C1	107.8 (3)	H30A—C30—H30C	109.5
C25—C10—C5	114.6 (3)	H30B—C30—H30C	109.5
C1—C10—C5	108.1 (3)	C3—O31—S34	120.2 (2)
C25—C10—C9	113.3 (3)	O32—S34—O33	119.9 (2)
C1—C10—C9	107.6 (3)	O32—S34—O31	103.60 (18)
C5—C10—C9	105.1 (3)	O33—S34—O31	110.48 (17)
C12—C11—C9	114.0 (3)	O32—S34—C35	108.1 (2)
C12—C11—H11A	108.7	O33—S34—C35	109.3 (2)
C9—C11—H11A	108.7	O31—S34—C35	104.37 (18)
C12—C11—H11B	108.7	C36—C35—C40	119.7 (4)
C9—C11—H11B	108.7	C36—C35—S34	121.1 (4)
H11A—C11—H11B	107.6	C40—C35—S34	119.1 (4)
C11—C12—C13	112.8 (3)	C35—C36—C37	120.2 (5)
C11—C12—H12A	109.0	C35—C36—H36	119.9
C13—C12—H12A	109.0	C37—C36—H36	119.9
C11—C12—H12B	109.0	C38—C37—C36	120.7 (5)
C13—C12—H12B	109.0	C38—C37—H37	119.7
H12A—C12—H12B	107.8	C36—C37—H37	119.7
C18—C13—C12	115.7 (3)	C39—C38—C37	118.3 (5)
C18—C13—C14	110.2 (3)	C39—C38—C41	120.8 (6)
C12—C13—C14	110.2 (3)	C37—C38—C41	120.8 (6)
C18—C13—H13	106.7	C38—C39—C40	122.3 (5)
C12—C13—H13	106.7	C38—C39—H39	118.8
C14—C13—H13	106.7	C40—C39—H39	118.8
C27—C14—C15	105.9 (3)	C39—C40—C35	118.6 (5)
C27—C14—C13	109.4 (3)	C39—C40—H40	120.7
C15—C14—C13	111.6 (3)	C35—C40—H40	120.7
C27—C14—C8	112.3 (3)	C38—C41—H41A	109.5
C15—C14—C8	110.6 (3)	C38—C41—H41B	109.5
C13—C14—C8	107.1 (3)	H41A—C41—H41B	109.5
C16—C15—C14	115.5 (3)	C38—C41—H41C	109.5
C16—C15—H15A	108.4	H41A—C41—H41C	109.5
C14—C15—H15A	108.4	H41B—C41—H41C	109.5
C16—C15—H15B	108.4	C28—O42—S45	117.7 (2)
C14—C15—H15B	108.4	O43—S45—O44	119.19 (18)
H15A—C15—H15B	107.5	O43—S45—O42	104.79 (17)
C15—C16—C17	112.1 (3)	O44—S45—O42	110.26 (17)
C15—C16—H16A	109.2	O43—S45—C46	110.50 (19)
C17—C16—H16A	109.2	O44—S45—C46	108.02 (19)
C15—C16—H16B	109.2	O42—S45—C46	102.85 (18)
C17—C16—H16B	109.2	C51—C46—C47	120.5 (4)
H16A—C16—H16B	107.9	C51—C46—S45	119.8 (3)
C16—C17—C22	116.1 (3)	C47—C46—S45	119.7 (4)
C16—C17—C28	110.8 (3)	C48—C47—C46	119.3 (4)
C22—C17—C28	109.3 (3)	C48—C47—H47	120.4
C16—C17—C18	107.9 (3)	C46—C47—H47	120.4
C22—C17—C18	102.0 (3)	C47—C48—C49	121.0 (4)
C28—C17—C18	110.5 (3)	C47—C48—H48	119.5
C13—C18—C17	112.2 (3)	C49—C48—H48	119.5
C13—C18—C19	119.8 (3)	C50—C49—C48	118.9 (4)
C17—C18—C19	104.6 (3)	C50—C49—C52	120.7 (4)
C13—C18—H18	106.5	C48—C49—C52	120.4 (4)
C17—C18—H18	106.5	C49—C50—C51	121.1 (4)
C19—C18—H18	106.5	C49—C50—H50	119.4
C20—C19—C21	112.1 (4)	C51—C50—H50	119.4
C20—C19—C18	117.2 (3)	C50—C51—C46	119.2 (4)
C21—C19—C18	103.2 (3)	C50—C51—H51	120.4
C20—C19—H19	108.0	C46—C51—H51	120.4
C21—C19—H19	108.0	C49—C52—H52A	109.5
C18—C19—H19	108.0	C49—C52—H52B	109.5
C29—C20—C19	121.2 (4)	H52A—C52—H52B	109.5
C29—C20—C30	120.7 (4)	C49—C52—H52C	109.5
C19—C20—C30	117.9 (4)	H52A—C52—H52C	109.5
C22—C21—C19	107.7 (3)	H52B—C52—H52C	109.5
			
C10—C1—C2—C3	−54.2 (5)	C12—C13—C18—C17	174.6 (3)
C1—C2—C3—O31	−178.8 (3)	C14—C13—C18—C17	−59.4 (4)
C1—C2—C3—C4	60.2 (5)	C12—C13—C18—C19	51.5 (5)
O31—C3—C4—C24	−52.9 (4)	C14—C13—C18—C19	177.4 (3)
C2—C3—C4—C24	67.5 (5)	C16—C17—C18—C13	63.3 (4)
O31—C3—C4—C23	64.8 (4)	C22—C17—C18—C13	−174.0 (3)
C2—C3—C4—C23	−174.8 (3)	C28—C17—C18—C13	−58.0 (4)
O31—C3—C4—C5	−179.2 (3)	C16—C17—C18—C19	−165.4 (3)
C2—C3—C4—C5	−58.7 (4)	C22—C17—C18—C19	−42.7 (4)
C3—C4—C5—C6	−174.3 (3)	C28—C17—C18—C19	73.4 (4)
C24—C4—C5—C6	60.8 (4)	C13—C18—C19—C20	−79.4 (5)
C23—C4—C5—C6	−59.2 (4)	C17—C18—C19—C20	153.8 (4)
C3—C4—C5—C10	55.2 (4)	C13—C18—C19—C21	156.9 (4)
C24—C4—C5—C10	−69.7 (4)	C17—C18—C19—C21	30.2 (4)
C23—C4—C5—C10	170.3 (3)	C21—C19—C20—C29	−102.1 (5)
C4—C5—C6—C7	161.9 (3)	C18—C19—C20—C29	138.9 (4)
C10—C5—C6—C7	−64.4 (4)	C21—C19—C20—C30	72.2 (5)
C5—C6—C7—C8	55.8 (4)	C18—C19—C20—C30	−46.8 (6)
C6—C7—C8—C26	74.7 (4)	C20—C19—C21—C22	−133.3 (4)
C6—C7—C8—C9	−46.2 (4)	C18—C19—C21—C22	−6.4 (4)
C6—C7—C8—C14	−165.2 (3)	C16—C17—C22—C21	155.0 (4)
C26—C8—C9—C11	62.5 (4)	C28—C17—C22—C21	−78.9 (4)
C7—C8—C9—C11	−179.5 (3)	C18—C17—C22—C21	38.0 (4)
C14—C8—C9—C11	−59.4 (4)	C19—C21—C22—C17	−19.7 (4)
C26—C8—C9—C10	−69.9 (4)	C16—C17—C28—O42	69.6 (4)
C7—C8—C9—C10	48.0 (4)	C22—C17—C28—O42	−59.5 (4)
C14—C8—C9—C10	168.2 (3)	C18—C17—C28—O42	−170.9 (3)
C2—C1—C10—C25	−75.4 (4)	C2—C3—O31—S34	113.4 (3)
C2—C1—C10—C5	49.1 (4)	C4—C3—O31—S34	−122.3 (3)
C2—C1—C10—C9	162.1 (3)	C3—O31—S34—O32	157.5 (3)
C6—C5—C10—C25	−63.3 (4)	C3—O31—S34—O33	27.9 (3)
C4—C5—C10—C25	69.2 (5)	C3—O31—S34—C35	−89.5 (3)
C6—C5—C10—C1	176.5 (3)	O32—S34—C35—C36	−121.6 (4)
C4—C5—C10—C1	−51.1 (4)	O33—S34—C35—C36	10.4 (4)
C6—C5—C10—C9	61.8 (4)	O31—S34—C35—C36	128.6 (4)
C4—C5—C10—C9	−165.7 (3)	O32—S34—C35—C40	54.7 (4)
C11—C9—C10—C25	−60.4 (4)	O33—S34—C35—C40	−173.3 (3)
C8—C9—C10—C25	70.4 (4)	O31—S34—C35—C40	−55.1 (4)
C11—C9—C10—C1	58.7 (4)	C40—C35—C36—C37	−3.1 (7)
C8—C9—C10—C1	−170.5 (3)	S34—C35—C36—C37	173.2 (4)
C11—C9—C10—C5	173.7 (3)	C35—C36—C37—C38	1.6 (8)
C8—C9—C10—C5	−55.5 (4)	C36—C37—C38—C39	1.1 (8)
C8—C9—C11—C12	52.1 (5)	C36—C37—C38—C41	−178.5 (5)
C10—C9—C11—C12	−174.6 (3)	C37—C38—C39—C40	−2.4 (8)
C9—C11—C12—C13	−49.4 (5)	C41—C38—C39—C40	177.2 (5)
C11—C12—C13—C18	−179.9 (3)	C38—C39—C40—C35	1.0 (7)
C11—C12—C13—C14	54.2 (4)	C36—C35—C40—C39	1.9 (7)
C18—C13—C14—C27	−68.5 (4)	S34—C35—C40—C39	−174.5 (3)
C12—C13—C14—C27	60.5 (4)	C17—C28—O42—S45	−149.0 (3)
C18—C13—C14—C15	48.3 (4)	C28—O42—S45—O43	−170.0 (3)
C12—C13—C14—C15	177.3 (3)	C28—O42—S45—O44	−40.6 (3)
C18—C13—C14—C8	169.5 (3)	C28—O42—S45—C46	74.4 (3)
C12—C13—C14—C8	−61.5 (4)	O43—S45—C46—C51	129.6 (4)
C26—C8—C14—C27	−178.4 (3)	O44—S45—C46—C51	−2.4 (4)
C7—C8—C14—C27	64.0 (4)	O42—S45—C46—C51	−119.0 (4)
C9—C8—C14—C27	−56.1 (4)	O43—S45—C46—C47	−53.1 (4)
C26—C8—C14—C15	63.5 (4)	O44—S45—C46—C47	174.9 (3)
C7—C8—C14—C15	−54.0 (4)	O42—S45—C46—C47	58.3 (4)
C9—C8—C14—C15	−174.2 (3)	C51—C46—C47—C48	1.8 (7)
C26—C8—C14—C13	−58.3 (4)	S45—C46—C47—C48	−175.6 (3)
C7—C8—C14—C13	−175.8 (3)	C46—C47—C48—C49	−1.1 (7)
C9—C8—C14—C13	64.0 (4)	C47—C48—C49—C50	−0.6 (7)
C27—C14—C15—C16	74.0 (4)	C47—C48—C49—C52	−179.5 (4)
C13—C14—C15—C16	−45.0 (4)	C48—C49—C50—C51	1.6 (7)
C8—C14—C15—C16	−164.1 (3)	C52—C49—C50—C51	−179.5 (4)
C14—C15—C16—C17	50.1 (5)	C49—C50—C51—C46	−0.9 (7)
C15—C16—C17—C22	−170.3 (4)	C47—C46—C51—C50	−0.8 (7)
C15—C16—C17—C28	64.3 (5)	S45—C46—C51—C50	176.5 (3)
C15—C16—C17—C18	−56.7 (4)		

### Hydrogen-bond geometry (Å, º)

**Table:**

*D*—H···*A*	*D*—H	H···*A*	*D*···*A*	*D*—H···*A*
C21—H21*B*···O43^i^	0.97	2.60	3.428 (5)	143
C26—H26*A*···O32^ii^	0.96	2.56	3.473 (5)	159
C28—H28*A*···O43^i^	0.97	2.39	3.244 (5)	147
C48—H48···O44^iii^	0.93	2.49	3.142 (5)	128

Symmetry codes: (i) *x*+1/2, −*y*+1/2, −*z*; (ii) −*x*+2, *y*−1/2, −*z*+1/2; (iii) *x*−1, *y*, *z*.
